# The influence of fluoxetine on the blood pressure: a meta-analysis of randomized controlled trials

**DOI:** 10.3389/fcvm.2026.1813209

**Published:** 2026-05-28

**Authors:** Deyun Huang, Wenjuan Wei, Kousalya Prabahar, Mohammad Safargar

**Affiliations:** 1 Department of Cardiovascular Medicine, The First People’s Hospital of Xiaoshan District, Hangzhou, China; 2 Department of Pharmacy Practice, Faculty of Pharmacy, University of Tabuk, Tabuk, Saudi Arabia; 3 Student Research Committee, Tabriz University of Medical Sciences, Tabriz, Iran

**Keywords:** blood pressure, CVD, diastolic blood pressure, fluoxetine, systolic blood pressure

## Abstract

**Background and aim:**

There are ongoing controversies regarding the impact of fluoxetine on blood pressure (BP), and a comprehensive analysis specifically addressing this relationship is lacking. Given the limited detail in existing literature on BP fluctuations during fluoxetine treatment, we conducted this meta-analysis to evaluate BP variations, providing clinically relevant insights.

**Methods:**

A structured and comprehensive review was conducted using systematic review and meta-analytic approaches. Only randomized controlled trials (RCTs) comparing fluoxetine with placebo were included. Exhaustive searches were performed across major scholarly databases, including Scopus, Web of Science, Embase, and PubMed/MEDLINE, covering all records up to July 28, 2025. Pooled estimates were calculated using the DerSimonian and Laird random-effects model, and results were reported as weighted mean differences (WMDs) with corresponding 95% confidence intervals (CIs).

**Results:**

Five RCT arms encompassing a total of 538 participants were included in the current meta-analysis. Fluoxetine treatment was associated with a statistically significant increase in systolic BP (SBP) (WMD: 7.47 mmHg; 95% CI: 0.14 to 14.80; *p* = 0.046) and diastolic BP (DBP) (WMD: 4.19 mmHg; 95% CI: 0.44 to 7.93; *p* = 0.028); however, these findings should be interpreted cautiously due to substantial heterogeneity and the limited number of included studies. Subgroup analyses based on treatment duration indicated that increases in SBP (WMD: 10.66 mmHg; 95% CI: 1.53 to 19.80; *p* = 0.022) and DBP (WMD: 6.65 mmHg; 95% CI: 4.30 to 8.99; *p* < 0.001) were more pronounced in trials lasting 12 weeks or less. No significant changes in BP were observed in studies with longer durations.

**Conclusions:**

While fluoxetine was associated with modest increases in SBP and DBP, these findings should not be interpreted as evidence supporting its clinical use for the management of hypotension or orthostatic hypotension. The included trials did not specifically enroll hypotensive populations, and the limited number of studies with substantial heterogeneity restricts the clinical applicability of this observation. Further clinical and experimental studies are warranted to better understand the relationship between fluoxetine and BP.

## Introduction

Systemic hypertension is a persistent increase in systemic arterial blood pressure (BP) and is a common condition that significantly contributes to both cardiovascular diseases (CVD) and mortality (1). Effective prevention and management of high BP are critical in reducing the risk and impact of these diseases, which in turn lowers associated mortality rates (2). However, inadequate management of hypertension remains one of the leading personal risk factors for global mortality, with a 10 mmHg increase in average systolic BP (SBP) linked to a 16% increased risk of CVD (3). Over the past three decades, the prevalence of hypertension among individuals aged 30 to 79 has doubled, from 331 million women to 626 million, and from 317 million men to 652 million by 2019 (4). Given these alarming statistics, studies have consistently shown that elevated BP increases the long-term risk of CVD. A clinical trial involving 1.25 million patients indicated that individuals with hypertension face a higher lifetime risk of CVD compared to those without hypertension (5).

Selective Serotonin Reuptake Inhibitors (SSRIs) are often the first-line treatment for depression, as they generally have a more favorable side-effect profile compared to other antidepressants. Among SSRIs, fluoxetine is widely prescribed for managing psychological disorders (6). SSRIs have demonstrated beneficial effects on CVD markers, including BP, blood glucose, and blood lipids (7, 8). Some studies have also noted a slight reduction in SBP and diastolic BP (DBP) with acute fluoxetine treatment compared to placebo (9). However, other research has found that fluoxetine does not significantly impact BP in depressed women without comorbidities (10). While SSRIs rarely cause cardiac death, they can result in side effects such as hypotension and mild bradycardia (11).

Controversial findings persist regarding the impact of fluoxetine on BP. Older patients on SSRIs are more likely to experience orthostatic hypotension compared to those not on SSRIs (12). Conversely, in younger individuals (ages 22–29), SSRIs are linked to higher BP (13). However, other studies have suggested that fluoxetine does not affect BP or heart rate (14).

Currently, there is limited information on the effect of fluoxetine on BP, and a comprehensive analysis addressing this issue is lacking. Given that the extent of BP fluctuations in relation to fluoxetine treatment remains underexplored, we conducted this meta-analysis to provide a clearer understanding of these variations, offering clinically relevant evidence.

## Material and methods

### Study design and literature search

To guide the conduct of this investigation, the research adhered to the methodological framework outlined by the PRISMA (Preferred Reporting Items for Systematic Reviews and Meta-Analyses) guidelines (15). A comprehensive and structured review of existing literature, incorporating both systematic review and meta-analytic approaches, was carried out through exhaustive searches across multiple scholarly databases, including Scopus, Web of Science, Embase, and PubMed/Medline, covering all records available up to July 28, 2025. To identify all relevant studies, a combination of controlled vocabulary (MeSH terms) and free-text keywords was utilized, specifically targeting the terms: (((Fluoxetine [MeSH Terms]) OR (Fluoxetine[tiab]))) AND ((((((((((Blood Pressure [Title/Abstract] OR Systolic Blood Pressure [Title/Abstract]) OR diastolic Blood Pressure [Title/Abstract]) OR “Blood Pressure"[Mesh]) OR SBP[Title/Abstract]) OR DBP[Title/Abstract]))))). Furthermore, manual screening of the reference sections of pertinent publications was undertaken to uncover any additional eligible studies not retrieved through the database search.

### Study selection

Study selection was performed independently by two reviewers to minimize bias. Both reviewers screened titles and abstracts, followed by full-text assessments of potentially eligible articles. Discrepancies were resolved through discussion, and if necessary, consultation with a third reviewer.

### Inclusion and exclusion criteria

To identify relevant RCT studies, the selection process was structured around the Population, Intervention, Comparator, and Outcome (PICO) framework. The inclusion criteria were as follows: (1) Population: individuals aged 18 years or older; (2) Intervention: administration of fluoxetine as the therapeutic modality; (3) Comparator: presence of a placebo-treated control group; and (4) Outcomes: availability of both mean values and standard deviations (SDs) for SBP and DBP, reported at baseline and at the conclusion of the intervention. Studies were excluded if they met any of the following conditions: absence of an appropriate control group, publication in languages other than English, presentation solely as conference abstracts, non-randomized controlled trial (non-RCT) design, or insufficient reporting of outcome data necessary for inclusion in the meta-analysis.

### Data extraction

Data extraction was independently performed by two reviewers, with any discrepancies resolved through discussion and consensus in consultation with the corresponding author. The following variables were systematically retrieved from each eligible study: the daily dosage of fluoxetine administered, duration of follow-up, year of publication, pre- and post-intervention mean values with corresponding standard deviations for the specified outcomes, name of the first author, health status of study participants, country in which the study was conducted, average age of participants, and total sample size.

### Risk of bias assessment

The methodological quality of the included trials was appraised using the Cochrane Risk of Bias 2 (RoB 2) tool. This instrument evaluates potential sources of bias across multiple domains, including biases arising from the randomization process, deviations from intended interventions, incomplete outcome data, outcome measurement procedures, and selective reporting of results (16).

### Statistical analysis

The pooled estimates of the outcomes were derived by calculating the differences in mean values using the DerSimonian and Laird random-effects model, which accounts for variability across studies. Results were presented as weighted mean differences (WMDs) accompanied by 95% confidence intervals (CIs). To evaluate between-study heterogeneity, Cochran's Q statistic was applied, and the extent of heterogeneity was quantified using the *I*^2^ statistic, with values interpreted as follows: low (<25%), moderate (26%–50%), and high (>50%) heterogeneity. A *p*-value less than 0.10 for the Q statistic was considered indicative of significant heterogeneity. An *a priori* subgroup analysis was planned to explore whether the daily fluoxetine dose, or intervention duration influenced the observed changes in BP outcomes. Publication bias across RCTs was assessed both visually using funnel plots and statistically through Egger's regression test, with a *p*-value <0.10 denoting significance (17). To evaluate the robustness of the findings, a sensitivity analysis was conducted by sequentially excluding each study and recalculating the overall effect estimates. For trials with multiple intervention arms sharing a common control group, we split the control group proportionally to avoid double counting, in accordance with Cochrane guidance. All statistical analyses were conducted using Stata software, version 15 (StataCorp, College Station, TX, USA), and a *p*-value below 0.05 was regarded as statistically significant.

## Results

### Study selection

**Figure 1:**
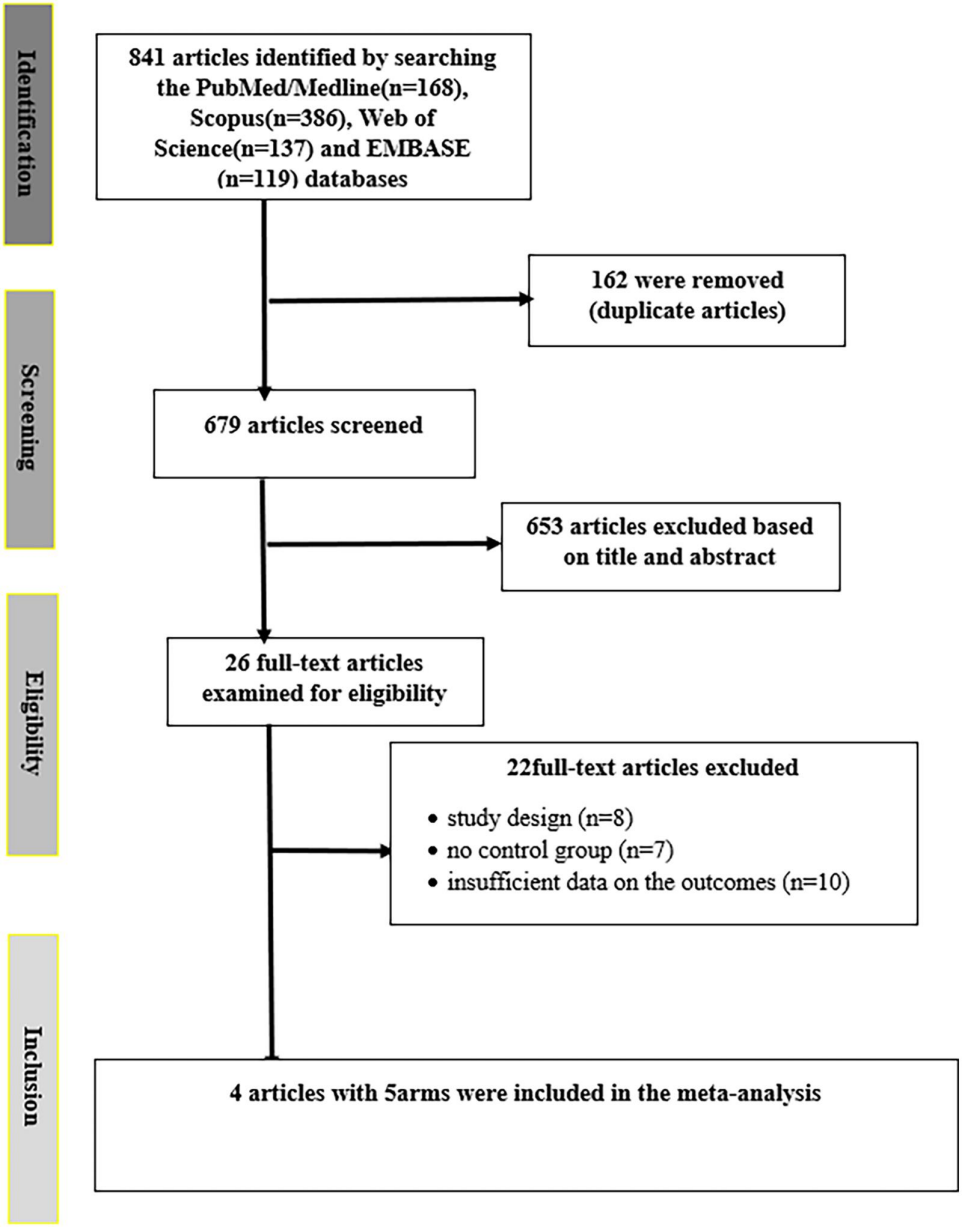
Flowchart depicting the study selection and inclusion processes for the present meta-analysis. RCT, randomized controlled trial(s).

Study selection was conducted independently by two reviewers to minimize bias. The initial search across electronic databases identified 841 records. After the removal of 162 duplicate entries, 679 unique articles remained for screening. Based on the evaluation of titles and abstracts, 653 studies were excluded for not meeting the inclusion criteria. Consequently, 26 full-text articles were assessed for eligibility. Following a thorough review, 4 studies comprising 5 RCT arms were deemed suitable for inclusion in the final meta-analysis (18–21) (Figure 1).

### Characteristics of the included studies

The RCTs incorporated in this analysis involved participants with a mean age spanning from 19.48 to 61.14 years. These trials were geographically diverse, having been undertaken in countries such as Brazil, the United States, the Netherlands, and China. Participants' body mass index (BMI) values fell within a range of 24.3 to 34.7 kg/m^2^. The selected studies were disseminated over a 25-year period, from 1993 through 2018. Intervention periods for fluoxetine administration varied considerably, extending from 6 weeks to a full year. Prescribed dosages of fluoxetine ranged between 20 mg and 80 mg per day. The study populations encompassed a broad spectrum of clinical and demographic profiles, including premenopausal obese women, individuals diagnosed with type 1 diabetes, men with central (abdominal) adiposity, healthy volunteers, and patients experiencing acute ischemic stroke. A comprehensive overview of the study attributes is presented in Table 1. The risk-of-bias assessment (Supplementary Figure S2) showed that all included studies were judged to be at low risk of bias across most domains. The only exception was attrition bias in the study by M. Visser, which was rated as unclear risk due to incomplete reporting of outcome data.

**Table 1:** Characteristics of the eligible RCTs.

Author	Year	Country	Population	Participants’ age (years)	Sample size: fluoxetine/placebo	BP measurement	Duration	Baseline BMI (kg/m^2^)	Fluoxetine (mg/day)	Control group
He, Y.	2018	China	Acute ishcemic stroke	61.14	202/202	Measured between 8 a.m. and 9 a.m. within 24 h of the patient's enrollment. The specific measurement device and the patient's position were not specified	90 days	≥30	20	Placebo
H. Suplicy	2014	Brazil	Obese premenopausal women	38.5	29/29	Measured as part of vital signs by research staff during morning visits, which took place after an overnight fast. The measurement device or the patient's position is not mentioned	52 weeks	34.7	20	Placebo
V. J. Briscoe(a)	2008	USA	type 1 diabetes patients	19.48	8/10	Measured noninvasively with a Dinamap (Critikon, Tampa, FL) every 10 min during a hypoglycemic clamp study conducted after an overnight fast. Patient position is not specified	6 weeks	25.3	20 mg/day during week 1 40 mg/day during week 2 60 mg/day during week 3 80 mg/day during weeks 4–6	Placebo
V. J. Briscoe(b)	2008	USA	healthy individuals	25.2	14/6	Measured noninvasively with a Dinamap (Critikon, Tampa, FL) every 10 min during a hypoglycemic clamp study conducted after an overnight fast. Patient position is not specified	6 weeks	24.3	20 mg/day during week 1 40 mg/day during week 2 60 mg/day during week 3 80 mg/day during weeks 4–6	Placebo
M. Visser	1993	The Netherlands	Abdominal overweight men	38.8 to 42.6	18/20	Measured with an oscillometric blood pressure computer (Boso-oscillomat, Jungingen, Germany) after the subject had been at rest in a supine position for 4 h. Measurements were taken on the left arm on two separate days during the baseline and endpoint weeks, and the two values were averaged	12 weeks	27.9	60	Placebo

Given the small number of included trials, this isolated uncertainty may still have influenced the pooled estimates. Therefore, the findings should be interpreted with appropriate caution, as even a single study with potential bias could disproportionately affect the overall results.

## Findings from the meta-analysis

### Impact of fluoxetine on SBP

**Figure 2:**
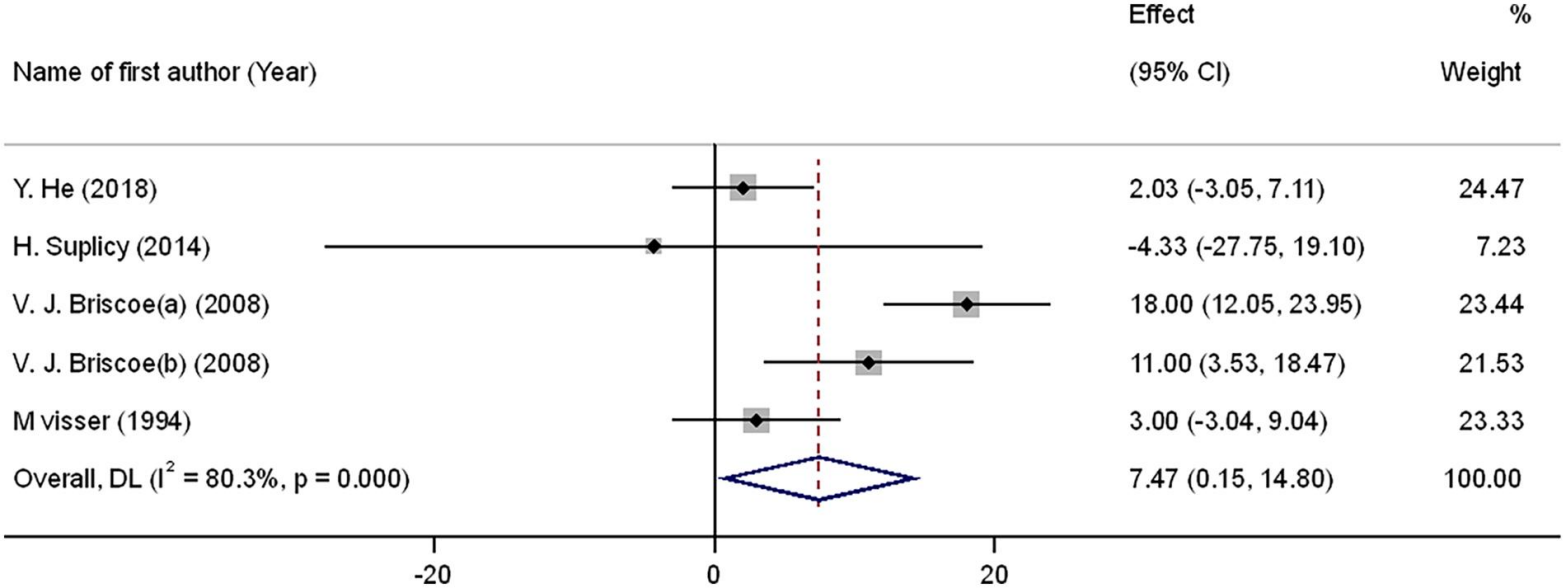
Forest plot of RCTs investigating the effects of fluoxetine on SBP. RCT, randomized controlled trial(s). SBP, systolic blood pressure; WMD, weighted mean difference; CI, confidence interval. Right side (positive WMD) favors fluoxetine (increase in SBP); left side (negative WMD).

The aggregated effect estimate derived from five RCT arms encompassing a total of 538 participants (fluoxetine group: *n* = 267; placebo group: *n* = 271) is illustrated in Figure 2. Treatment with fluoxetine was associated with a statistically significant elevation in SBP, with a WMD of 7.47 mmHg (95% CI: 0.14 to 14.80; *p* = 0.046). However, substantial heterogeneity was detected across the included studies (*I*^2^ = 80%, *p* < 0.001). Subgroup analyses based on treatment duration revealed that the rise in SBP was more pronounced in trials lasting 12 weeks or less (WMD: 10.66 mmHg, 95% CI: 1.53 to 19.80; *p* = 0.022), while no significant change was observed in trials exceeding 12 weeks (WMD: 1.74 mmHg, 95% CI: −3.22 to 6.71; *p* = 0.491) (see Supplementary Figure S1).

### Impact of fluoxetine on DBP

**Figure 3:**
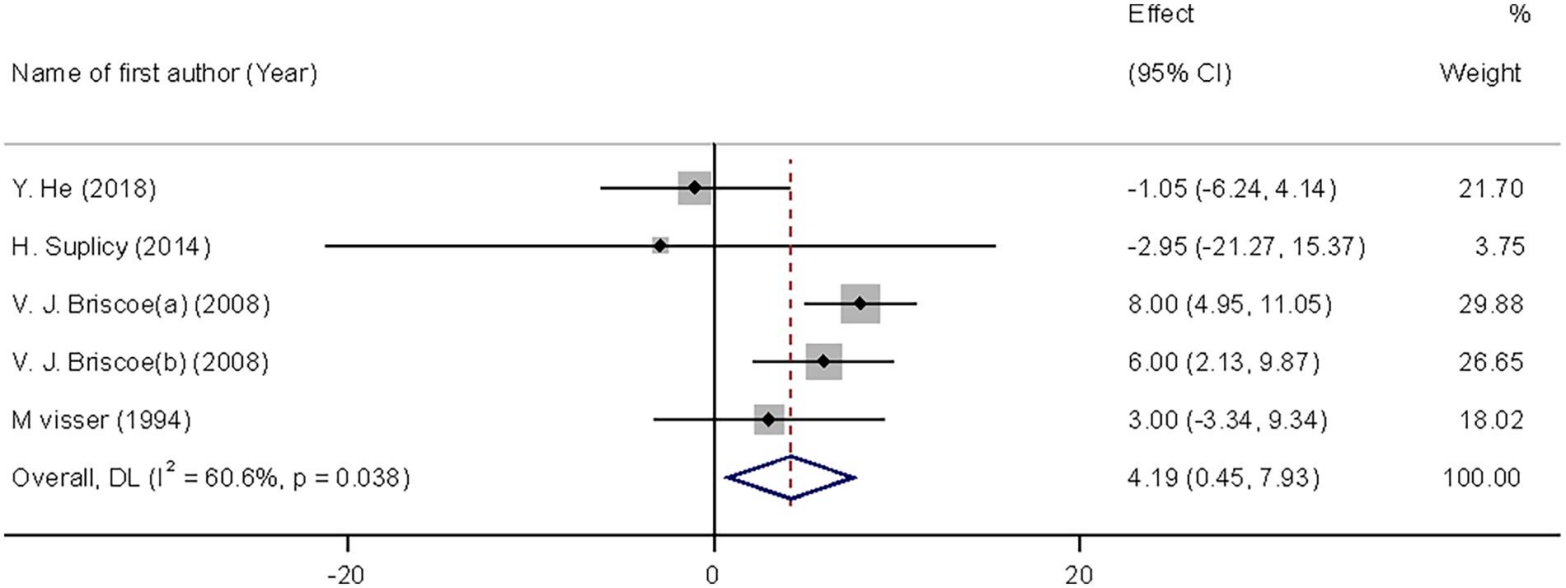
Forest plot of RCTs investigating the effects of fluoxetine on DBP. RCT, randomized controlled trial(s); DBP, diastolic blood pressure; WMD, weighted mean difference; CI, confidence interval.

Figure 3 presents the pooled estimate for changes in DBP based on the same five RCT arms (fluoxetine: *n* = 267; placebo: *n* = 271). Fluoxetine administration resulted in a statistically significant increase in DBP, with a WMD of 4.19 mmHg (95% CI: 0.44 to 7.93; *p* = 0.028). Moderate heterogeneity was observed among the included trials (*I*^2^ = 60%, *p* = 0.028). In stratified analyses, trials with a duration of 12 weeks or less showed a marked increase in DBP (WMD: 6.65 mmHg, 95% CI: 4.30 to 8.99; *p* < 0.001), whereas longer interventions (>12 weeks) did not yield significant changes (WMD: −1.19 mmHg, 95% CI: −6.18 to 3.80; *p* = 0.640) (see Supplementary Figure S1).

### Sensitivity analysis

To assess the robustness of the meta-analysis outcomes, sensitivity analyses were conducted by systematically excluding each individual RCT arm. The results of the analysis did not materially change the direction or significance of the pooled estimates (see Supplementary Figure S2).

## Discussion

In this meta-analysis, fluoxetine treatment was associated with a statistically significant increase in both SBP and DBP; however, substantial heterogeneity limits the certainty of these findings. Evidence regarding the relationship between SSRIs and BP remains inconsistent and appears to be influenced by patient characteristics and clinical context. Previous studies have reported no significant association between SSRIs and BP in patients with cardiovascular disease, while others have demonstrated reductions in DBP among individuals with hypertension and psychiatric disorders (22, 23). Additionally, SSRIs have been linked to orthostatic hypotension, particularly in older adults at risk of falls (24).

The variability observed in our analysis may partly reflect the clinical diversity of the included populations, which ranged from premenopausal obese women and individuals with type 1 diabetes to healthy volunteers and stroke patients. Pooling such heterogeneous groups may obscure population-specific effects and limit generalizability, given likely differences in autonomic regulation, vascular function, baseline BP, and pharmacodynamic responses to fluoxetine.

Real-world data further illustrate the complexity of this relationship. Humbert et al. reported variable rates of SSRI-associated hypertension across pharmacovigilance databases, with fluoxetine implicated in a subset of cases (25). Aslam et al. reported that SSRIs are associated with elevated nighttime SBP and DBP, potentially leading to adverse cardiac effects (26). Fluoxetine has been shown to activate the sympathetic nervous system in the heart and adrenal medulla by increasing the expression of tyrosine hydroxylase and dopamine beta-hydroxylase genes, resulting in higher noradrenaline production and stimulating vasopressin release, both of which contribute to increased BP (27).

The treatment duration of fluoxetine also affects its association with BP. Our subgroup analyses based on treatment duration indicated that the rise in SBP was more pronounced in trials lasting 12 weeks or less (*p* = 0.022), whereas no significant change was observed in studies exceeding 12 weeks (*p* = 0.491). Similarly, trials of 12 weeks or less showed a significant increase in DBP (*p* < 0.001), while longer interventions (>12 weeks) did not produce significant changes (*p* = 0.640). Proposed mechanisms for the influence of SSRIs on BP include: (a) Serotonin can either constrict or dilate blood vessels depending on the integrity of the endothelial lining. SSRIs may elevate serotonin levels, potentially causing vasoconstriction and raising BP when endothelial integrity is compromised. Conversely, SSRIs could lower BP when the endothelial lining is intact (28). (b) SSRIs may enhance sympathetic activation, contributing to an increase in nocturnal BP (29). However, given the small number of included trials and limited statistical power, these subgroup findings should be considered exploratory and hypothesis-generating rather than definitive evidence of a duration-dependent effect.

Long-term fluoxetine use can alter vascular sympathetic adrenergic responses by modulating pre-synaptic mechanisms. It increases beta-arrestin activity and reduces calcium stores in vascular smooth muscle cells, leading to diminished vasoconstriction. This may reduce peripheral vascular resistance and lower BP, potentially contributing to orthostatic hypotension, a side effect commonly associated with prolonged fluoxetine use (30).

This meta-analysis has several limitations. The small number of included RCTs and modest sample sizes limit statistical power and restrict exploration of heterogeneity. Clinical heterogeneity across study populations further complicates interpretation, as differing baseline characteristics may influence BP responses to fluoxetine. Additionally, some trials contributed multiple intervention arms without appropriate adjustment for shared control groups, which may have inflated effect estimates; such adjustments were not feasible due to limited reporting. Variability in BP measurement methods, including differences in posture, device type, cuff size, and timing, represents a major source of potential bias and may have contributed to both within- and between-study heterogeneity. Furthermore, subgroup and meta-regression analyses based on population characteristics were not feasible due to insufficient data. Finally, publication bias was not formally assessed because of the limited number of studies.

Overall, these limitations underscore the need for cautious interpretation. Future well-designed, adequately powered RCTs with standardized BP measurement protocols and more homogeneous populations are required to clarify the effects of fluoxetine on BP.

## Conclusion

While fluoxetine was associated with modest increases in SBP and DBP, these findings should not be interpreted as evidence supporting its clinical use for the management of hypotension or orthostatic hypotension. The included trials did not specifically enroll hypotensive populations, and the limited number of studies with substantial heterogeneity restricts the clinical applicability of this observation. Further clinical and experimental studies are needed to better understand the impact of fluoxetine on BP.

## Data Availability

The original contributions presented in the study are included in the article/Supplementary Material, further inquiries can be directed to the corresponding author.

## References

[1] Mills KT Stefanescu A He J. The global epidemiology of hypertension. Nat Rev Nephrol. (2020) 16(4):223–37. 10.1038/s41581-019-0244-2 32024986 PMC7998524

[2] Carey RM Muntner P Bosworth HB Whelton PK. Prevention and control of hypertension: jACC health promotion series. J Am Coll Cardiol. (2018) 72(11):1278–93. 10.1016/j.jacc.2018.07.008 30190007 PMC6481176

[3] Alharran AM Alzayed MM Jamilian P Prabahar K Kamal AH Alotaibi MN Impact of magnesium supplementation on blood pressure: an umbrella meta-analysis of randomized controlled trials. Curr Ther Res. (2024) 101:100755. 10.1016/j.curtheres.2024.100755 39280209 PMC11401110

[4] Zhou B Carrillo-Larco RM Danaei G Riley LM Paciorek CJ Stevens GA Worldwide trends in hypertension prevalence and progress in treatment and control from 1990 to 2019: a pooled analysis of 1201 population-representative studies with 104 million participants. Lancet. (2021) 398(10304):957–80. 10.1016/S0140-6736(21)01330-1 34450083 PMC8446938

[5] Rapsomaniki E Timmis A George J Pujades-Rodriguez M Shah AD Denaxas S Blood pressure and incidence of twelve cardiovascular diseases: lifetime risks, healthy life-years lost, and age-specific associations in 1· 25 million people. Lancet. (2014) 383(9932):1899–911. 10.1016/S0140-6736(14)60685-1 24881994 PMC4042017

[6] Edinoff AN Akuly HA Hanna TA Ochoa CO Patti SJ Ghaffar YA Selective serotonin reuptake inhibitors and adverse effects: a narrative review. Neurol Int. (2021) 13(3):387–401. 10.3390/neurolint13030038 34449705 PMC8395812

[7] Bellon A Nguyen K. Selective serotonin reuptake inhibitors and risk reduction for cardiovascular disease in patients with schizophrenia: a controversial but promising approach. World J Psychiatry. (2021) 11(7):316. 10.5498/wjp.v11.i7.316 34327124 PMC8311507

[8] Liu D Guo T Peng Q Velu P Prabahar K Safargar M The effect of Fluoxetine on lipid profiles in overweight or obese individuals: a systematic review and meta-analysis of randomized controlled trials. Diabetes Res Clin Pract. (2025) 222:112040. 10.1016/j.diabres.2025.112040 39978642

[9] Amsterdam JD Garcia-Espana F Fawcett J Quitkin FM Reimherr FW Rosenbaum JF Blood pressure changes during short-term fluoxetine treatment. J Clin Psychopharmacol. (1999) 19(1):9–14. 10.1097/00004714-199902000-00004 9934937

[10] Beyazyüz M Albayrak Y Eğilmez OB Albayrak N Beyazyüz E. Relationship between SSRIs and metabolic syndrome abnormalities in patients with generalized anxiety disorder: a prospective study. Psychiatry Investig. (2013) 10(2):148. 10.4306/pi.2013.10.2.148 PMC3687049 23798963

[11] Nezafati MH Eshraghi A Vojdanparast M Abtahi S Nezafati P. Selective serotonin reuptake inhibitors and cardiovascular events: a systematic review. J Res Med Sci. (2016) 21(1):66. 10.4103/1735-1995.189647 27904611 PMC5122239

[12] Briggs R Carey D McNicholas T Claffey P Nolan H Kennelly SP The association between antidepressant use and orthostatic hypotension in older people: a matched cohort study. J Am Soc Hypertens. (2018) 12(8):597–604.e1. 10.1016/j.jash.2018.06.002 29937420

[13] Crookes DM Demmer RT Keyes KM Koenen KC Suglia SF. Depressive symptoms, antidepressant use, and hypertension in young adulthood. Epidemiology. (2018) 29(4):547–55. 10.1097/EDE.0000000000000840 29629939 PMC5980764

[14] Graff DW Williamson KM Pieper JA Carson SW Adams KF Cascio WE Effect of fluoxetine on carvedilol pharmacokinetics, CYP2D6 activity, and autonomic balance in heart failure patients. J Clin Pharmacol. (2001) 41(1):97–106. 10.1177/00912700122009746 11225566

[15] Moher D Liberati A Tetzlaff J Altman DG. Reprint—preferred reporting items for systematic reviews and meta-analyses: the PRISMA statement. Phys Ther. (2009) 89(9):873–80. 10.1093/ptj/89.9.873 19723669

[16] Higgins JPT Altman DG Gotzsche PC Juni P Moher D Oxman AD The cochrane Collaboration’s tool for assessing risk of bias in randomised trials. Br Med J. (2011) 343:d5928. 10.1136/bmj.d5928 22008217 PMC3196245

[17] Egger M Smith GD Schneider M Minder C. Bias in meta-analysis detected by a simple, graphical test. Br Med J. (1997) 315(7109):629–34. 10.1136/bmj.315.7109.629 9310563 PMC2127453

[18] He Y Cai Z Zeng S Chen S Tang B Liang Y Effect of fluoxetine on three-year recurrence in acute ischemic stroke: a randomized controlled clinical study. Clin Neurol Neurosurg. (2018) 168:1–6. 10.1016/j.clineuro.2018.02.029 29494855

[19] Suplicy H Boguszewski CL dos Santos CMC do Desterro de Figueiredo M Cunha DR Radominski R. A comparative study of five centrally acting drugs on the pharmacological treatment of obesity. Int J Obes. (2014) 38(8):1097–103. 10.1038/ijo.2013.225 24287940

[20] Briscoe VJ Ertl AC Tate DB Dawling S Davis SN. Effects of a selective serotonin reuptake inhibitor, fluoxetine, on counterregulatory responses to hypoglycemia in healthy individuals. Diabetes. (2008) 57(9):2453–60. 10.2337/db08-0236 18567822 PMC2518497

[21] Visser M Seidell JC Koppeschaar HPF Smits P. No specific effect of fluoxetine treatment on fasting glucose, insulin, lipid levels, and blood pressure in healthy men with abdominal obesity. Obes Res. (1994) 2(2):152–9. 10.1002/j.1550-8528.1994.tb00641.x 16353616

[22] Zhang L Li G Liu M. A meta-analysis on the association between SSRIs and blood pressure in patients with CVD and depression. J Affect Disord. (2023) 340:181–8. 10.1016/j.jad.2023.08.032 37557986

[23] Razavi Ratki SK Seyedhosseini S Valizadeh A Rastgoo T Tavakkoli R Golabchi A Can antidepressant drug impact on blood pressure level in patients with psychiatric disorder and hypertension? A randomized trial. Int J Prev Med. (2016) 7(1):26. 10.4103/2008-7802.174891 26941927 PMC4755252

[24] Gaxatte C Faraj E Lathuillerie O Salleron J Deramecourt V Pardessus V Alcohol and psychotropic drugs: risk factors for orthostatic hypotension in elderly fallers. J Hum Hypertens. (2017) 31(4):299–304. 10.1038/jhh.2013.82 24048292

[25] Humbert X Fedrizzi S Chrétien B Sassier M Bagheri H Combret S Hypertension induced by serotonin reuptake inhibitors: analysis of two pharmacovigilance databases. Fundam Clin Pharmacol. (2019) 33(3):296–302. 10.1111/fcp.12440 30489655

[26] Aslam N Memon S Wadei H Niazi S. Effect of serotonin reuptake inhibitors (SSRIS) and serotonin-norepinephrine reuptake inhibitors (SNRIS) on blood pressure variability using 24 h ambulatory blood pressure monitoring. In: de Borst M, editor. Nephrology Dialysis Transplantation. Oxford OX2 6DP, England: Oxford Univ Press Great Clarendon ST (2020) 35(3):gfaa142.P0150. 10.1093/ndt/gfaa142.P0150

[27] Lazartigues E Brefel-Courbon C Bagheri H Costes S Gharib C Tran M Fluoxetine-induced pressor response in freely moving rats: a role for vasopressin and sympathetic tone. Fundam Clin Pharmacol. (2000) 14(5):443–51. 10.1111/j.1472-8206.2000.tb00426.x 11129084

[28] Dimoula A Fotellis D Aivalioti E Delialis D Polissidis A Patras R Off-target effects of antidepressants on vascular function and structure. Biomedicines. (2021) 10(1):56. 10.3390/biomedicines10010056 35052735 PMC8773150

[29] Calvi A Fischetti I Verzicco I Belvederi Murri M Zanetidou S Volpi R Antidepressant drugs effects on blood pressure. Front Cardiovasc Med. (2021) 8:704281. 10.3389/fcvm.2021.704281 34414219 PMC8370473

[30] Pereira CA Rodrigues FL Ruginsk SG Zanotto CZ Rodrigues JA Duarte DA Chronic treatment with fluoxetine modulates vascular adrenergic responses by inhibition of pre-and post-synaptic mechanisms. Eur J Pharmacol. (2017) 800:70–80. 10.1016/j.ejphar.2017.02.029 28216049

