# Supplementary material for: The influence of fluoxetine on the blood pressure: a meta-analysis of randomized controlled trials
Source: Front Cardiovasc Med. 2026 May 28;13:1813209. doi: 10.3389/fcvm.2026.1813209 (PMC13253682; doi:10.3389/fcvm.2026.1813209)
Supplement: Supplementary file 1 [file Image1.pdf]

Supplementary Figure 1. subgroup analyses

RCT, randomized controlled trial(s). WMD, weighted mean difference. CI, confidence interval. SBP, systolic blood pressure. DBP, diastolic blood pressure.

Length of intervention (weeks)

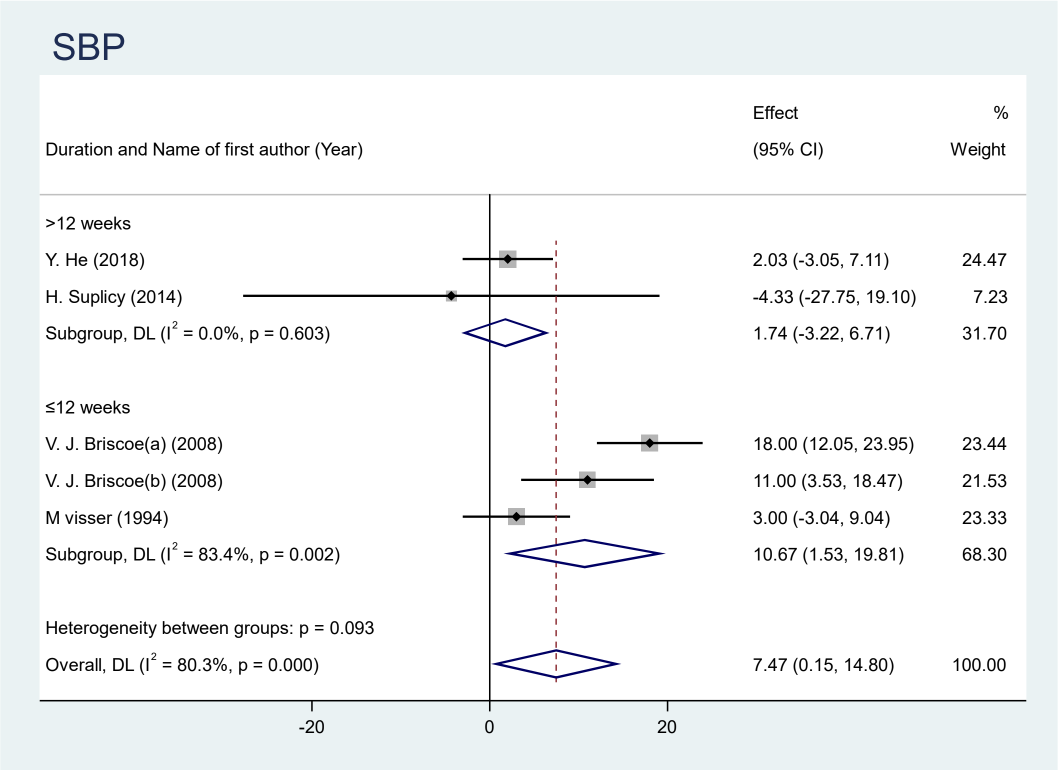

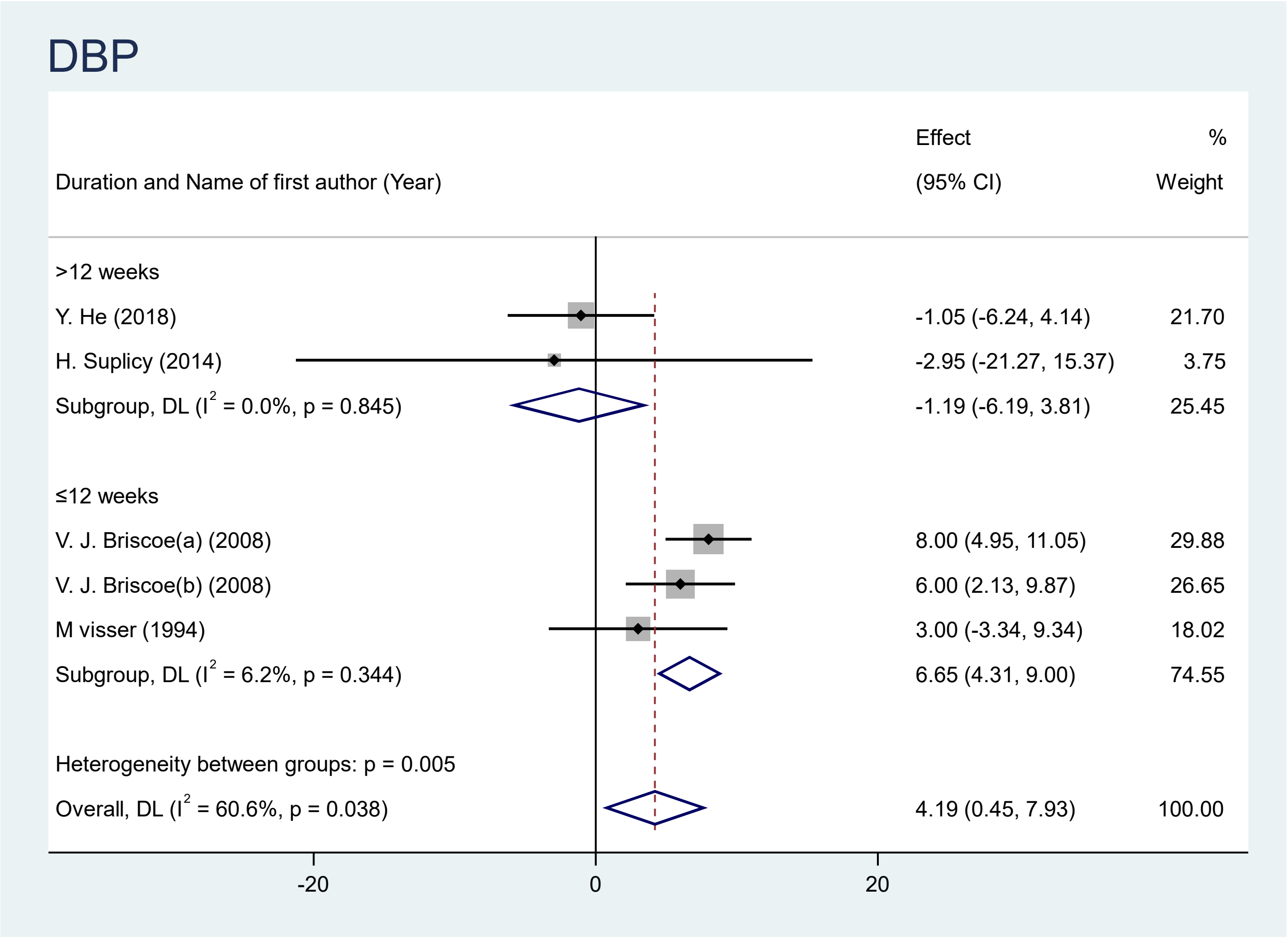

Fluoxetine dosage (mg/day)

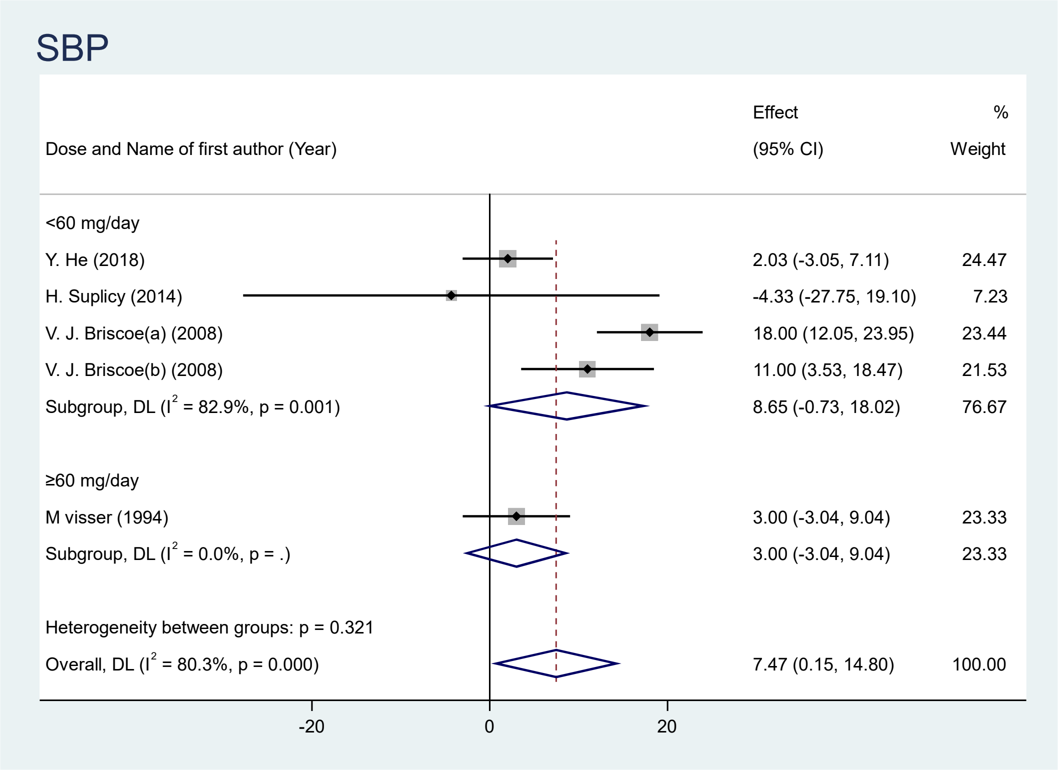
SBP
Effect
(95% CI)
%
Weight
Dose and Name of first author (Year)
<60 mg/day
Y. He (2018) 2.03 (-3.05, 7.11) 24.47
H. Suplicy (2014) -4.33 (-27.75, 19.10) 7.23
V. J. Briscoe(a) (2008) 18.00 (12.05, 23.95) 23.44
V. J. Briscoe(b) (2008) 11.00 (3.53, 18.47) 21.53
Subgroup, DL (I^2 = 82.9%, p = 0.001) 8.65 (-0.73, 18.02) 76.67
≥60 mg/day
M visser (1994) 3.00 (-3.04, 9.04) 23.33
Subgroup, DL (I^2 = 0.0%, p = .) 3.00 (-3.04, 9.04) 23.33
Heterogeneity between groups: p = 0.321
Overall, DL (I^2 = 80.3%, p = 0.000) 7.47 (0.15, 14.80) 100.00
-20
0
20

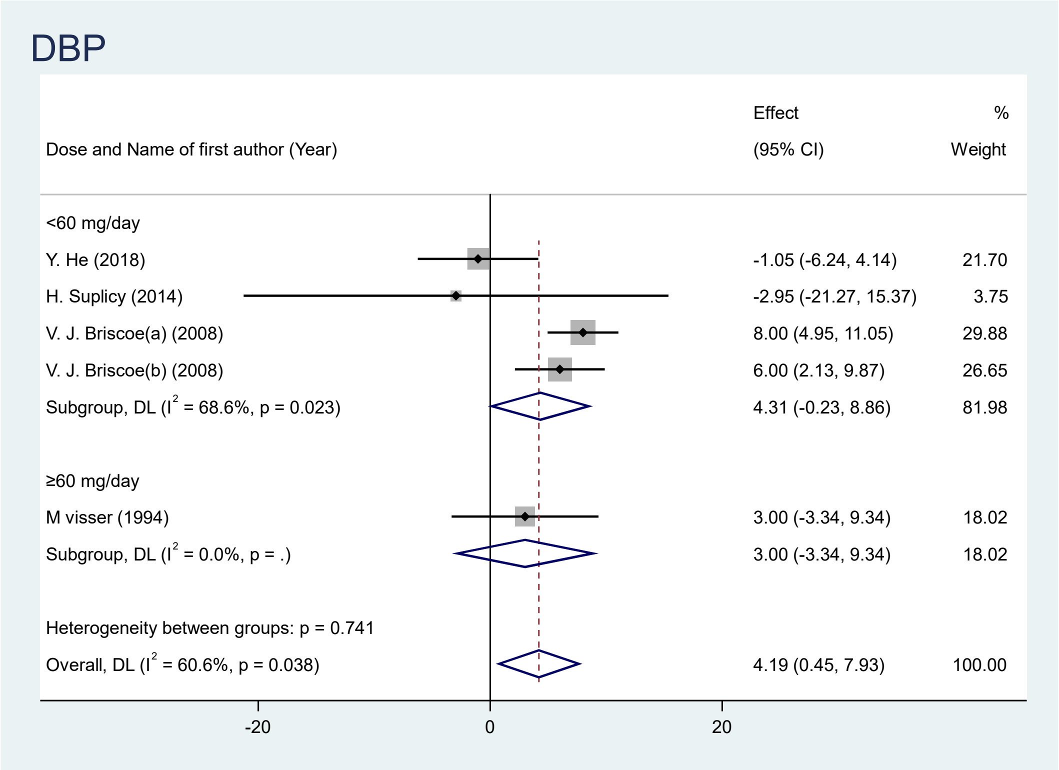
DBP
Effect
(95% CI)
%
Weight
Dose and Name of first author (Year)
<60 mg/day
Y. He (2018) -1.05 (-6.24, 4.14) 21.70
H. Suplicy (2014) -2.95 (-21.27, 15.37) 3.75
V. J. Briscoe(a) (2008) 8.00 (4.95, 11.05) 29.88
V. J. Briscoe(b) (2008) 6.00 (2.13, 9.87) 26.65
Subgroup, DL (I^2 = 68.6%, p = 0.023) 4.31 (-0.23, 8.86) 81.98
≥60 mg/day
M visser (1994) 3.00 (-3.34, 9.34) 18.02
Subgroup, DL (I^2 = 0.0%, p = .) 3.00 (-3.34, 9.34) 18.02
Heterogeneity between groups: p = 0.741
Overall, DL (I^2 = 60.6%, p = 0.038) 4.19 (0.45, 7.93) 100.00
-20
0
20
