# Supplementary material for: The influence of fluoxetine on the blood pressure: a meta-analysis of randomized controlled trials
Source: Front Cardiovasc Med. 2026 May 28;13:1813209. doi: 10.3389/fcvm.2026.1813209 (PMC13253682; doi:10.3389/fcvm.2026.1813209)
Supplement: Supplementary file 2 [file Image2.pdf]

Supplementary Figure 2. Sensitivity analysis**.**

SBP, systolic blood pressure. DBP, diastolic blood pressure. CI, confidence interval.

A) SBP

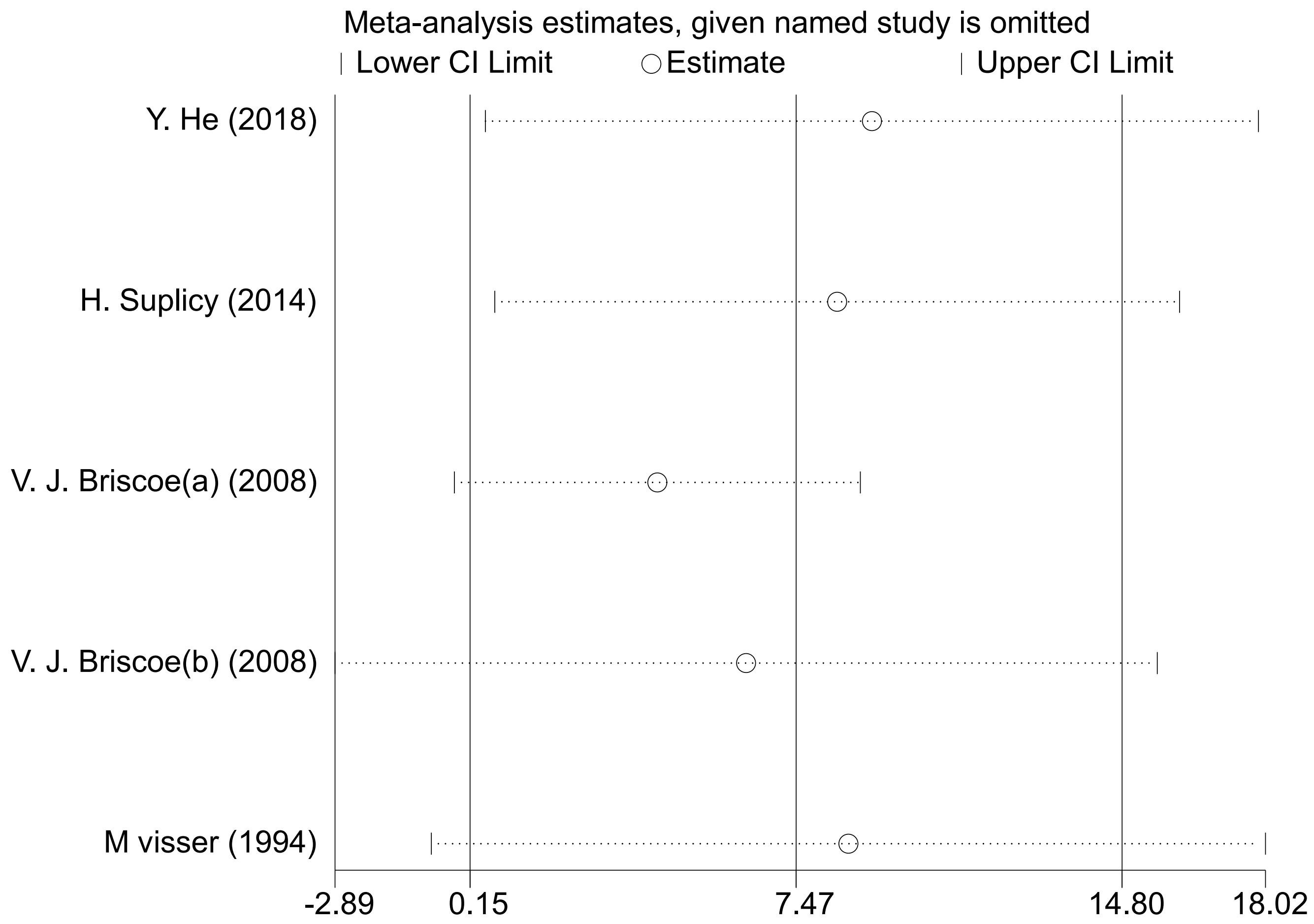

b) DBP

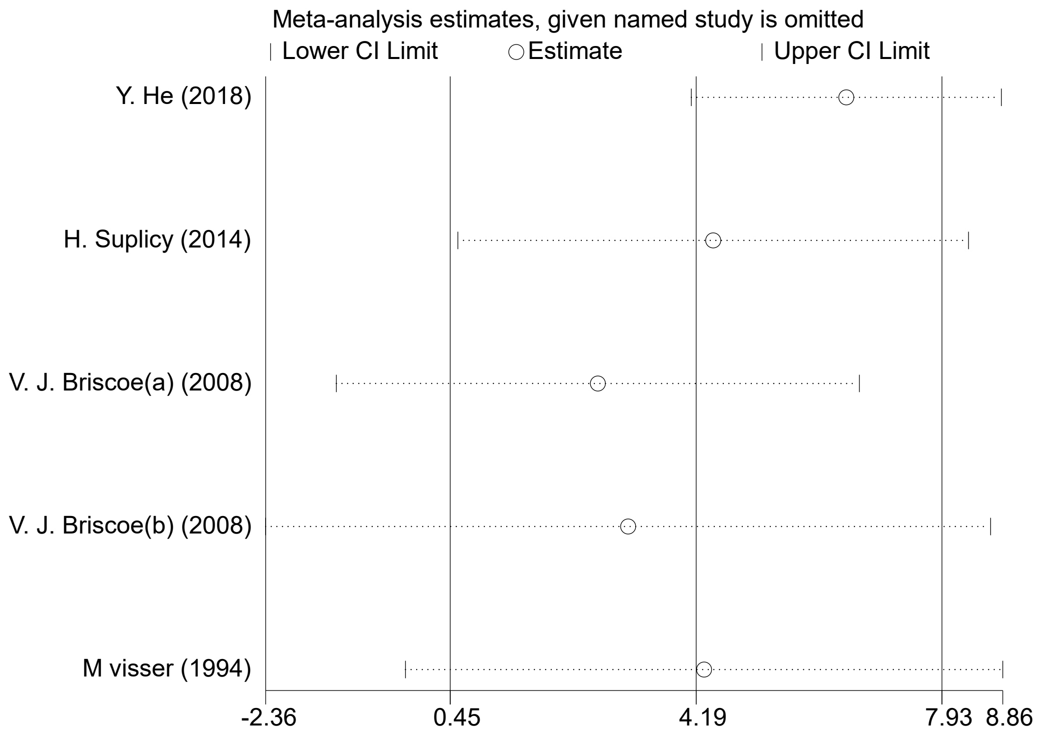

Meta-analysis estimates, given named study is omitted
Lower CI Limit
Estimate
Upper CI Limit
Y. He (2018)
H. Suplicy (2014)
V. J. Briscoe(a) (2008)
V. J. Briscoe(b) (2008)
M visser (1994)
-2.36
0.45
4.19
7.93
8.86
