# Supplementary material for: The influence of fluoxetine on the blood pressure: a meta-analysis of randomized controlled trials
Source: Front Cardiovasc Med. 2026 May 28;13:1813209. doi: 10.3389/fcvm.2026.1813209 (PMC13253682; doi:10.3389/fcvm.2026.1813209)
Supplement: Supplementary file 3 [file Table1.docx]

Supplementary Table 1. Search strategy.

| PubMed/MEDLINE | Scopus | Web of Science | Embase |
| --- | --- | --- | --- |
| (((Fluoxetine[MeSH Terms]) OR (Fluoxetine[tiab]))) AND ((((((((((Blood Pressure[Title/Abstract] OR Systolic Blood Pressure[Title/Abstract]) OR diastolic Blood Pressure[Title/Abstract]) OR "Blood Pressure"[Mesh]) OR SBP[Title/Abstract]) OR DBP[Title/Abstract])))) | ( TITLE-ABS-KEY ( fluoxetine ) ) AND ( TITLE-ABS-KEY ( "blood pressure" OR "systolic blood pressure" OR "diastolic blood pressure" OR sbp OR dbp ) ) | TS=(fluoxetine) AND TS=("blood pressure" OR "systolic blood pressure" OR "diastolic blood pressure" OR SBP OR DBP) | ('fluoxetine'/exp OR fluoxetine:ti,ab) AND ('blood pressure'/exp OR 'systolic blood pressure':ti,ab OR 'diastolic blood pressure':ti,ab OR 'blood pressure':ti,ab OR sbp:ti,ab OR dbp:ti,ab) |
