# Supplementary material for: The influence of fluoxetine on the blood pressure: a meta-analysis of randomized controlled trials
Source: Front Cardiovasc Med. 2026 May 28;13:1813209. doi: 10.3389/fcvm.2026.1813209 (PMC13253682; doi:10.3389/fcvm.2026.1813209)
Supplement: Supplementary file 4 [file Table2.docx]

**Supplementary Table 2:** The risk of bias in studies included in this study

|  | Random Sequence Generation (selection bias) | Allocation concealment (selection bias) | Blinding of participants and personnel (performance bias) | Blinding of outcome assessment (detection bias) | Incomplete outcome data (attrition bias) | Selective reporting (reporting bias) | Other bias | AHRQ standards |
| --- | --- | --- | --- | --- | --- | --- | --- | --- |
| He, Y. |  |  |  |  |  |  |  | Good |
| H. Suplicy |  |  |  |  |  |  |  | Good |
| V. J. Briscoe(a) |  |  |  |  |  |  |  | Good |
| V. J. Briscoe(b) |  |  |  |  |  |  |  | Good |
| M. Visser |  |  |  |  | 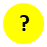 |  |  | Fair |
